# Males conditionally inseminate at three female body locations according to female mating history and female maturity status in a squid

**DOI:** 10.1038/s41598-024-62062-7

**Published:** 2024-05-22

**Authors:** Kamrun Naher Azad, Md. Nur E. Alam, Makoto Nagata, Satoshi Tomano, Hiroki Ono, Kyoko Sugai, Noritaka Hirohashi

**Affiliations:** 1 Graduate School of Natural Science and Technology, Shimane University, Matsue, Japan; 2 Department of Aquaculture, Bangladesh Agricultural University, Mymensingh, Bangladesh; 3 Faculty of Pharmacy, Osaka Medical and Pharmaceutical University, Takatsuki, Japan; 4 Atmosphere and Ocean Research Institute, University of Tokyo, Kashiwa, Japan; 5 Marine Biological Science Section, Education and Research Center for Biological Resources, Faculty of Life and Environmental Science, Shimane University, Matsue, Japan

**Keywords:** Cephalopod reproduction, Alternative reproductive tactics, Postcopulatory sexual selection, Condition-dependence, Sperm competition, Sperm allocation, Behavioural ecology, Animal behaviour

## Abstract

In some squids, such as those in the family Loliginidae, upon copulation, females receive and store male-delivered sperm capsules, spermatangia, at two different body locations: the buccal membrane and the distal end of the oviduct. This insemination site dimorphism is associated with alternative reproductive strategies. However, in *Loliolus sumatrensis*, a species of Loliginidae, the females possess three insemination sites: buccal membrane (BM), basal left IV arm (ARM) and lateral head behind the left eye (EYE), therefore we studied such the unusual phenomena. We developed microsatellite markers and genotyped the paternity of each spermatangium on three sites. We found multiple paternity at every single site and simultaneous usage of all three sites by a few males. The seasonal dynamics of a population in the Seto Inland Sea revealed a set priority for the initial use of insemination sites as BM, followed by ARM and then EYE, whereas the maximum number of stored spermatangia was greater in EYE > ARM > BM. Female maturity status was correlated with the usage pattern of insemination sites but not with the number of stored spermatangia at any insemination site. These results suggest that a male squid inseminates at different locations according to female mating history and female maturity status.

## Introduction

To achieve reproductive success, males often change their mating behaviours in response to environmental, intersexual, and intrasexual conditions. Such behavioural plasticity during mating by males is conceptualized by the theory of evolutionary stable strategy under promiscuous circumstances. For example, sperm allocation, a type of male-oriented mate choice, involves cost-effective distribution of reproductive resources. Because even though a substantial number of sperm are produced by males, sperm are regarded as a limited reproductive resource^1–3^. Thus, the theory predicts that males allocate their ejaculate expenditure to females in response to future male mating opportunities^4^ or the reproductive conditions of the focal females^5^ such as fecundity^6,7^ or promiscuity^8–12^, in favor of a cost–benefit trade-off within the context of promiscuous mating. If so, males should immediately evaluate individual females and decide the extent of sperm expenditure to invest in each female, in accordance with their estimated reproductive value. Sperm allocation is known to occur in a wide range of taxa, including insects^4,8,9^, crustaceans^13^, fish^11,14^ and birds^12,15^.

Another case in which males can switch their mating behaviors is observed in animals that employ alternative reproductive tactics (ARTs)^16^. In ARTs, males that are recessive in physical, social, or reproductive status among same-sex competitors adopt different, unusual, and often sneaky approaches to gain access to mates or gametes, and sneaker males tend to invest more ejaculates in quality and quantity than bourgeois males^17–19^. However, in commonly observed ARTs, males make their decisions ontogenetically during development or growth, and these decisions are often associated with male size dimorphism^20,21^.

In some squids, such as the family Loliginidae, ARTs also occur in relation to male size dimorphism; smaller and larger males copulate and transfer spermatophores to females at the buccal membrane (BM) where seminal receptacle is located or near the oviduct, respectively, resulting in two different sperm deposition sites within a female^22–25^. Choosing one of two insemination sites by males largely relies on the relative size difference between a mating pair—males that are smaller than mating females inseminate spermatophores at the BM, whereas males that are larger than a female tend to transfer spermatophores in the vicinity of the oviduct^23^. Hence, it is believed that each male chooses only one of the two insemination sites on a focal female at the time of copulation^23,26^.

While extending our ART studies to various loliginid species, we found that *Loliolus sumatrensis* females have three distinctive sperm deposition sites: the buccal membranes (BM), basal areas of the left IV arm (ARM), and the lateral head behind the left eye (EYE). Driven by curiosity about this unusual phenomenon in this family, we attempted to identify the usage patterns of these sites in relation to the mating history, maturity, fecundity, and growth indices of female individuals. In addition, microsatellite markers were developed and used to measure the level of multiple paternity of the deposited spermatangia at each sperm insemination site. We found that although all sites were used by multiple males, there were males who unexpectedly used more than two insemination sites per female. This could be regarded as an unusual form of sperm allocation, where males may change the insemination site within a female in response to the current sperm storage status and fecundity. We explore the possible advantages of each site under circumstances in which both sexes have relatively greater mating opportunities before females initiate egg spawning.

## Results

### Insemination at three different female locations by monomorphic males

**Figure 1:**
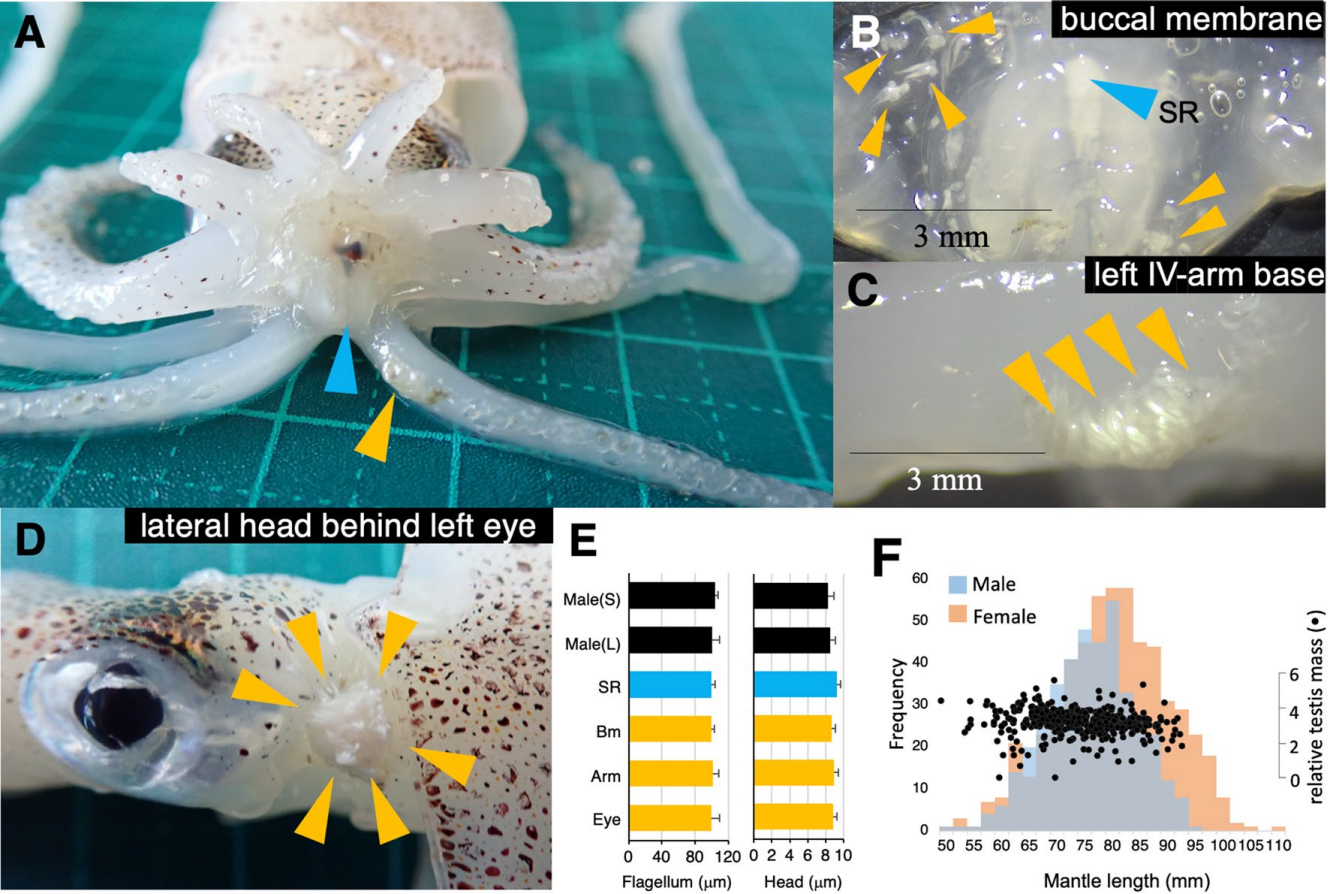
*L. sumatrensis* possess three insemination sites within a female. **A**–**D**, representative photographs showing female spermatangia-attachment sites: the buccal area around the mouth (**A**) containing the sperm storage organ, seminal receptacle (*bule arrowheads* in **A** and **B**); spermatangia (*yellow arrowheads*) attached to buccal membrane (**B**), left IV arm (**C**) and lateral head behind the left eye (**D**). **E**, Sperm flagellum and head lengths. Sperm were collected from small males (*Male*(*S*)), large males (*Male*(*L*)), female seminal receptacles (*SR*), buccal membrane (*Bm*), left IV arm (*Arm*) and lateral head behind the left eye (*Eye*). The mantle length ranges for smaller males (*Male*(*S*)) and larger males (*Male*(*L*)) were 55–65 mm and 85–95 mm, respectively. **F**, The mantle length distributions of adult males and females collected during the fishery season and relative testis mass across variance in body size.

We first conducted morphological and anatomical investigations of adult squids collected from the Seto Inland Sea, Japan. The species was identified as *L. sumatrensis* by the dentition morphology of the largest sucker rings in the tentacles and III arms^27^, and thereafter by mitochondrial genomic DNA sequencing (electronic supplementary material, Fig. S1). We found that spermatangia were attached to females at three different sites: the buccal membrane (Fig. 1A,B; BM), basal left IV arm (Fig. 1C; ARM), and lateral head behind the left eye (Fig. 1D; EYE). With DNA barcoding and a species-specific PCR-based assay for the spermatangia at the three sites, we found no evidence of heterospecific cross-insemination (electronic supplementary materials, Fig. S1). Moreover, no significant differences in sperm size (flagellum and head) were observed among spermatangia at the three insemination sites (GLMM, *P* > 0.05; Fig. 1E). The mature male individuals showed monomodal size distribution with similar relative testis mass (Fig. 1F), suggesting the absence of male dimorphism in body size that is commonly observed in other squids with ARTs^26^. Of note, the average relative testis mass was the highest (3.18 ± 0.75, n = 397) in the loliginid family examined so far^28^, suggesting a highly promiscuous mating mode in *L. sumatrensis*.

### Genotyping-based validation for the site-dependent promiscuity and multiple-site usage by single males

**Figure 2:**
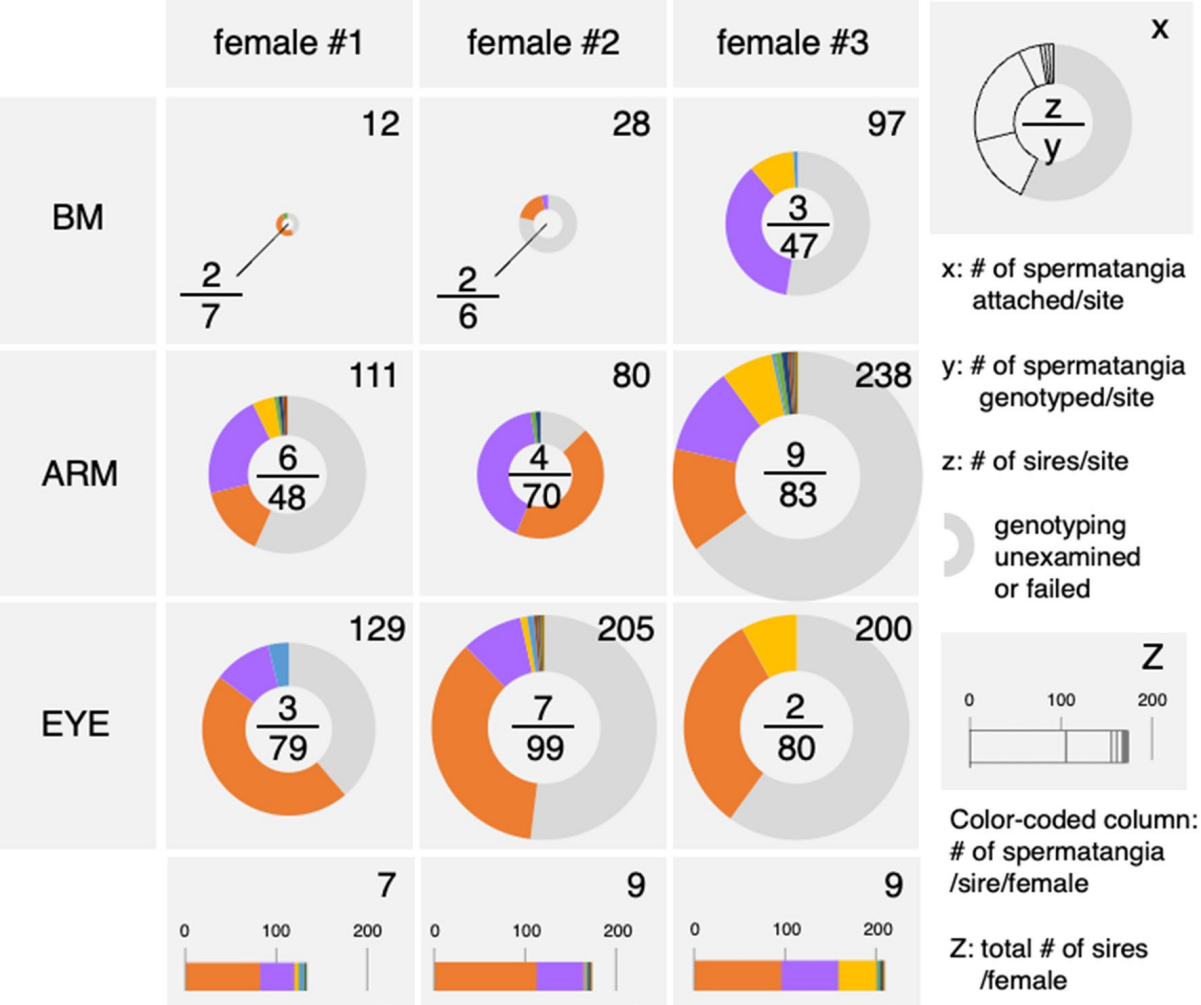
The insemination site-dependent spermatangium number and sire number. Genotyping of attached spermatangia was carried out from three females. The number of spermatangia attached to each insemination site was shown (x, *top-right in each panel with a pie chart*), of which the number of spermatangia successfully isolated/genotyped was indicated as the denominator (y) and sire number was indicated as the numerator (z). The lower panels represent the total number of sires (Z, *top-right*) and total number of spermatangia/sire/female which was color-coded in the stacked columns. The size of each pie chart corresponds with the total number of attached spermatangia/site.

To address the level of multiple paternity (promiscuity) at each insemination site, we developed microsatellite markers (Table 1, *Materials and Methods*), by which every spermatangium attached to each site was genotyped (Fig. 2, electronic supplementary materials, Table S2). We found that the actual number of sires per site was higher at ARM (6.3 ± 2.5, n = 3) than at the other two sites (BM, 2.3 ± 0.5; EYE, 4.0 ± 2.6, n = 3), but there were no significant differences in number of sires among the three sites (GLMM, *P* > 0.05). Surprisingly, a few males inseminated simultaneously at multiple (two or three) sites on the same female, and these small numbers of sires were found to have the major part of the paternity share (95.4% ± 2.7%) in the total attached spermatangia (Fig. 2).

**Table 1:** Information and characterization of newly developed microsatellite markers.

Locus	Repeat	Forward sequence	Reverse sequence	N	Na	Ho	He	Fis	Pid
luy1738	(AGA)24	ATGCGGAAAGGTGTGATTGT	TTTATGCCCCTCTTCCTCCT	46	22	0.826	0.922	0.153	0.014
luy0529	(CTT)25/(TTA)17	TAACTGCAATGCCCAATCTG	CAAACACGCTGGCGATATAA	43	30	0.628	0.939	0.342*	0.007
luy4288	(ATA)18	AAGACTCCAATGAAAGACCACT	CAGAAGCCACAAATCGCCTA	47	22	0.766	0.932	0.188*	0.009
luy3099	(TTA)21	CCATTTAAACACGAGATGCAA	CCAGTTAACGTTGGTGTGAAAA	44	14	0.905	0.942	0.001	0.019

N, sample size; Na, number of alleles; Ho, observed heterozygosity; He, expected heterozygosity; Fis, fixation index; *, significance of departure from Hardy–Weinberg Equilibrium (P < 0.01); Pid, probability of genetic identity.

### A set priority for initial use of insemination sites within a female

**Figure 3:**
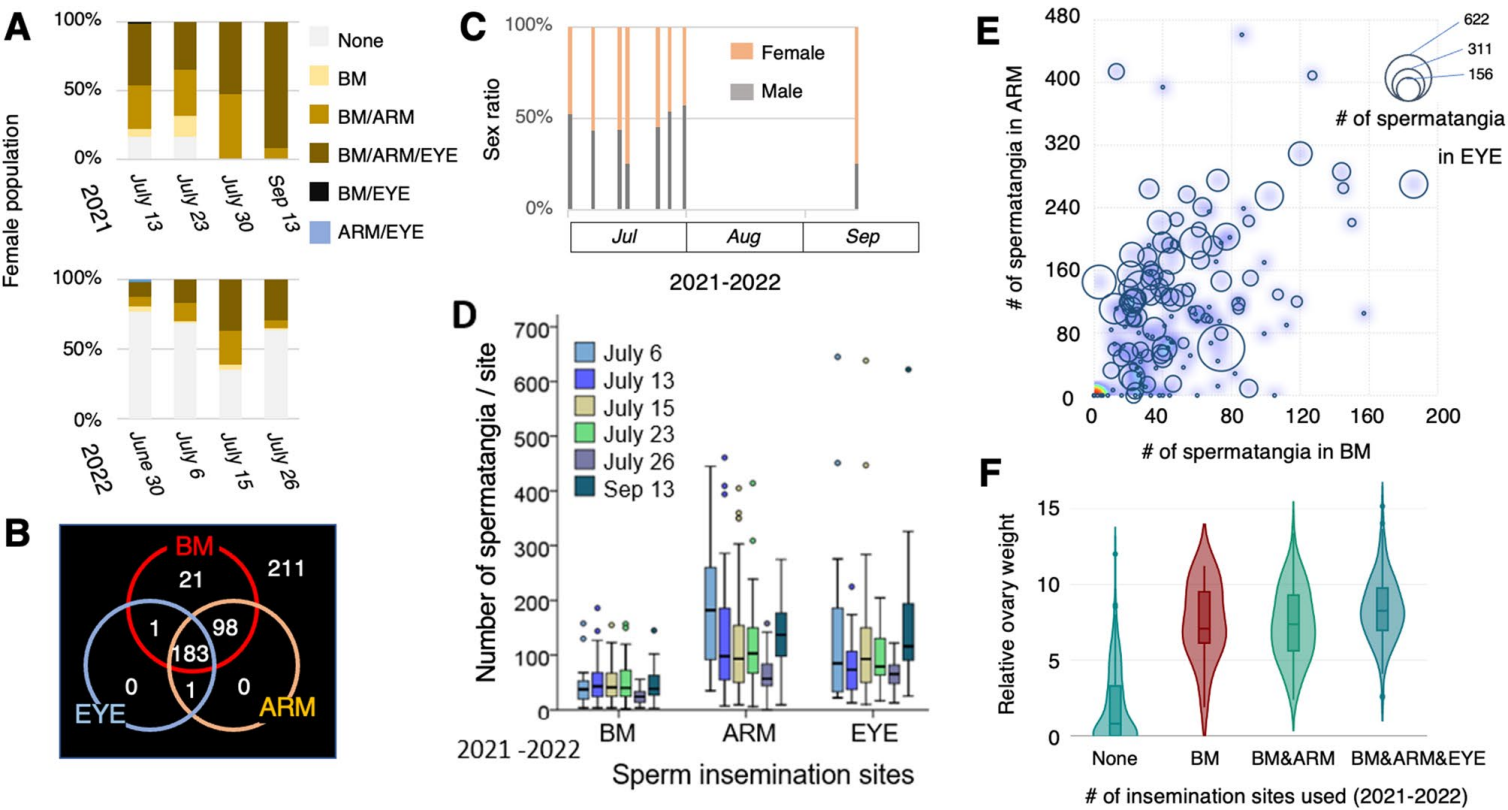
Seasonal dynamics of the insemination pattern and quantity in *L. sumatrensis*. (**A)** Six insemination patterns were identified in females during two-year consecutive fishery seasons (2021–2022). (**B)** A venn diagram showing the number of females classified into above six patterns. (**C)** Sex ratio was measured at each fishing day. (**D)** The number of spermatangia attached to each insemination site within a female in different fishing days. (**E)** Numerical distributions of attached spermatangia per site within a female. Darkened *blue* indicates a higher plot density (number of individuals). (**F)** Female individuals with different insemination patterns (BM, BM&ARM and BM&ARM&EYE) are compared in terms of relative ovary weight. The graph represents boxplots (quartiles) merged with violin plots.

We classified the usage patterns of the three insemination sites and presented them as the proportions of females with these patterns for each fishing day (Fig. 3A). There were six different patterns, and at the end of the fishery season (September 13, 2021), 91.8% of the females had spermatangia at all three sites. The analysis of usage patterns allows us to speculate the sequence of the initial use of insemination sites on a female, i.e., first appearance of spermatangia at the BM and then ARM followed by EYE (Fig. 3B, electronic supplementary materials, Table S3), which was further supported by the statistical analysis of rank cases (electronic supplementary materials, Table S4).

To explain seasonal changes in insemination patterns and an observed set priority for initial usage, we raised three scenarios: (1) the operational sex ratio becomes more male-biased as the season progresses; therefore, males use all three sites in response to increased male-male competition; (2) sperm-storing capacity in each site is limited, but the mating season continues; therefore, late-coming males must choose other sites; or (3) females become fully mature and fecund, resulting in more attractive for males to mate.

The sex ratio was almost unbiased or female-biased in the late fishery season (%male on September 13 = 25.3; Fig. 3C), suggesting that scenario (1) is unlikely. In a population collected throughout the two seasons, the number of spermatangia attached to the BM was significantly smaller than that in the other two sites (GLMM, Tukey–Kramer test, P < 0.0001, BM, 46.2 ± 33.1; ARM, 126.4 ± 94.9; EYE, 116.3 ± 111.3; Fig. 3D). At each insemination site, the number of spermatangia were substantially high in individual variance (Fig. 3E; electronic supplementary materials, Fig. S2A) and significantly different among fishing days (GLM; BM, *P* = 0.0217; ARM, *P* = 0.0014; EYE, *P* = 0.0022), however, we found no consistent trends (increasing or decreasing) of the spermatangium quantity throughout the season (Fig. 3D). Furthermore, the multiple regression model showed that the spermatangium number at any of the insemination sites was not much affected by the spermatangium number at other sites (BM: R^2^ = 0.15, F_2,241_ = 22.66, P < 0.0001; ARM: R^2^ = 0.24, F_2,241_ = 38.61, P < 0.0001; EYE: R^2^ = 0.12, F_2,241_ = 17.73, P < 0.0001; Fig. 3E), indicating that the observed set priority for the initial insemination (BM → ARM → EYE) was not due to the full occupancy of preferred insemination sites; therefore, scenario (2) was unlikely.

However, the mated females were significantly different from the unmated females in their relative ovary weight (GLMM, Tukey–Kramer test, P < 0.0001, Fig. 3F). Moreover, the relative ovary weight was significantly higher in females inseminated at all three sites than two or fewer sites (LMM, Tukey Kramer test, P = 0.0002, Fig. 3F), supporting the scenario (3). Furthermore, the multiple regression models incorporating ML, BW, ACC, ovary weight (OW) and SITE to explain variation in the total number of attached spermatangia/female (TOTAL) were statistically significant (R^2^ = 0.34, F_5,238_ = 26.23, P < 0.0001, electronic supplementary materials, Table S5). Notably, however, the total and site-dependent number of attached spermatangia were not correlated with the growth and maturity status of female (electronic supplementary materials, Table S5; Fig. S2). Lastly, we evaluated the costs/benefits of utilization of each insemination site. Based on hypothetical conditions (see *Materials and Methods*), we assessed and ranked the subjects that could potentially influence fertilization success of the deposited spermatangia (Table 2).

**Table 2:** Measurements, estimation and ranking of subjects that potentially influence fertilization success.

Subjects	Site of insemination		
	BM	ARM	EYE
Distance from egg deposition site	Proximal	Sub-proximal	Distal
Distance from seminal receptacle	Proximal	Sub-proximal	Distal
Order in the first use	First	Second	Third
Mean (maximum) number of spermatangia attached	46.2 ± 33.1 (186)	126.4 ± 94.9 (461)	116.3 ± 111.3 (645)
Estimated placement size for spermatangia attachment	Smaller	Intermediate	Larger
Estimated lifetime of spermatangia to be attached	Shorter	Longer	Longer
Number of paternity detected (n = 3)	2–3	4–9	2–7

## Discussion

Because sperm are a limited reproductive resource, males in polygamous species may be adapted to allocate their ejaculate expenditure to females effectively^5^. Thus, sperm allocation by an individual male occurs in response to sociosexual environments that could be influenced by the status of females, rivals, and that male’s own condition^29^. To achieve maximum reproductive success, males must evaluate sociosexual environments through the perception of visual, chemical, acoustic, and tactile cues^30–32^. One of the key elements that could impact on male mating behaviour regarding sperm allocation is the risk of sperm competition, which is a powerful evolutionary driver^33^. Sperm competition also drives developmental, morphological, and behavioural plasticity in relation to sex. ARTs are the best examples. Meta analysis on ARTs literature across a wide range of animal taxa identified that sperm produced by parasitic (sneaker) males are greater in number and swimming speed than bourgeois males^34^. ARTs are commonly associated with developmentally regulated male dimorphism^21^. Thus, sperm allocation could be developed as the consequences of ARTs. In contrast, conditional ARTs occurring in some organisms are of particular interest when addressing the behavioral correlation between sperm allocation and ARTs.

In the loliginid squid *L. sumatrensis,* we found three insemination sites (BM, ARM, and EYE) located discontinuously on a female. This differs from the cases in other species of this family, where two separate locations—the buccal membrane and oviduct—are alternatively used by males along with different mating strategies ^23,25^. A remarkable feature that is common in most squid ARTs is the linkage between the insemination sites and sperm traits: sperm inseminated at BM have longer flagella, whereas sperm inseminated near the oviduct have shorter flagella^35^. Thus, in squid, the morphological traits of sperm are generally considered to be adaptive to the insemination environments and their associated sperm storage modes^36^.

However, some loliginid squid species also show context-dependent ARTs^22,37–40^, where males flexibly change mating tactics in response to relative size differences between mating pairs^22,39^ , which resulted in attenuated sperm dimorphism^40^. In any case, males must choose the designated areas of female body locations, because the insemination site greatly influences the fertilization success (but see^41–44^).

The current study presents a sharp contrast to well-known squid ARTs. First, both male body size and sperm flagellum length showed monomodal distributions (Fig. 1E,F). Second, there was a set order for the first use of insemination sites (BM → ARM → EYE). Third, nevertheless males use all three sites simultaneously (sperm allocation within a female) in some cases. We wondered what factors make a change in male mating behaviours (insemination sites) even during copulation. First, we considered the possibility that because male-male competition at mating is so intense, males must use other insemination sites in favor of reducing the sperm competition risk.

We considered and measured some factors that might have impacts on fertilization of the deposited sperm at each insemination site (Table 2). Taking these conditions into account, we speculated that BM is the most favorable site for insemination by *L. sumatrensis* males owing to its proximity to both the egg deposition site and the seminal receptacle (Table 2). In agreement with this, BM was chosen as the first among the three sites (Fig. 3B). However, the mean and maximum numbers of spermatangia attached to BM were smaller than those attached to the other two sites (Fig. 3D,E), despite having some vacant space for insemination at BM. The lower number of spermatangia at the BM might be associated with the seminal receptacle being progressively enriched with sperm, although the dynamics of sperm storage in the seminal receptacle is unknown. It is interesting to hypothesize that males can sense the vacant status of the seminal receptacle either directly or indirectly. In fact, cephalopods have more neurons in their arms than in their brains and perceive chemotactile sensation through their arms, suggesting that the arms play more perceptive roles than just being used as flexible actuators. Notably, the spermatangium remnants were frequently observed at BM (electronic supplementary materials, Fig. S3) but not at the other sites, suggesting the occurrence of rapid attachment-detachment turnover of the spermatangia. Thus, sperm at BM might be used immediately for fertilization (proximal time points to egg spawning) or translocated to the seminal receptacle for longer storage. The latter case can explain the occurrence of BM utilization in the first order because the vacant seminal receptacles (virgin females) are the most favorable for first-mating males to use^45^. We assumed that because the area of EYE is larger and nearer to the oviduct opening than those of BM or ARM, EYE is preferred by males who can invest more sperm resources to females with higher fecundity (greater in relative OW, Fig. 3F). However, it is difficult to envisage how the sperm located at EYE could reach fertilization. One speculation would be that because EYE site lies at the region where seawater enters the mantle cavity and the oviduct lies on the left side of the female, the influx of water might bring the sperm attached at the left side of EYE and results in fertilization. In contrast, ARM is located in the area capable of flexible movements around the mouth, which may allow the ARM-deposited spermatangia to become proximal to eggs or the seminal receptacle during egg capsule manipulation between the arms before deposition on the substrate. Although ARM exhibited the highest average paternity number (n = 3, Table 2), further genotyping with more specimens is required to evaluate the site-dependent promiscuity. Given that BM, ARM and EYE have mutually distinct sperm-storing characteristics (Table 2), this can be explained by the concept of a polymorphic fitness equilibrium^46^ in which reproductive success of sperm at each sperm-deposition site changes dynamically depending on the current overall utilization state. In other words, at the individual level, once all insemination sites within a female have begun to be used, forthcoming inseminations by other males could occur anywhere based on the most favorable site under the current circumstances.

This is essentially analogous to, but apparently different from, a well-recognized sperm allocation theory in which males strategically allocate sperm to each female in response to sperm competition risk^2,8,9,11,12^, the number of available mates^4,5,15,47^, and female quality or fecundity^6,7,13,14,48^. In the case of squid, however, sperm allocation occurs within a female.

We hereto disregarded the likelihood of female active involvement in the process of male behavioural decisions, simply because female squids are generally promiscuous and lack conspicuous sexual dimorphism. However, given that the firefly squid, *Watasenia scintillans* exhibits highly monoandrous insemination albeit its operational sex ratio is extremely male-biased^28^, we therefore cannot rule out the possibility that male inseminations are under female control. In addition, we cannot rule out the possibility that seasonal changes in insemination patterns may reflect seasonal movements of females with different reproductive behaviours. To resolve these problems, future studies require captive experimental settings in which male mating behaviours to females with different mating histories can be observed, and the paternity success rate by sperm at each insemination site can be analyzed. In conclusion, we found that female sexual experiences and the resulting occupancy rate at insemination sites might provide cues for subsequent males to choose their insemination site(s). Hence, we propose that alternative reproductive tactics can arise even in species that lack both direct male-male combat for mating and male body size polymorphism.

## Materials and methods

### Animal collection and species identification

The squid, *Loliolus sumatrensi*s were purchased from a fisherman as dead animals around the Shodo Island in the Seto Inland Sea, Japan during the fishery season (July–September) of this species in 2021 and 2022 and transported as ice-cold (for sperm size measurements) or frozen specimens (for DNA analysis). During this period, we obtained 917 individuals (397 males and 520 females) on eight different fishing days. Individual data are summarized in electronic supplementary materials, Table S1. *Loliolus japonica* were obtained from the same fisherman in May 2022. Fishing points were informed from a fisherman who caught these squids. Squids were killed by a fisherman as part of routine commercial food. First, species identification was carried out based on overall morphology and more specifically on largest sucker ring dentitions in the tentacles and the third arms^27^, allowing us to distinguish from closely-related, morphologically-similar species such as *Loliolus japonica* or *L. beka.* However, the morphology of sucker rings in *L. sumatrensis* was undistinguishable from that of *L. uyii*, therefore cytochrome c oxidase I (*COI*) DNA sequencing was carried out for representative specimens with genomic DNAs isolated from mantle tissues, testes as well as the spermatangia, and universal primers that potentially amplify *Loliolus* species (electronic supplementary materials, Fig. S1A); *Loliolus* universal *COI* forward primer: 5′- CAATGTAGTAGTAACTGCTCACGG -3′, *Loliolus* universal *COI* reverse primer: 5′- GCTCCTAAAATAGAAGAAATACCA -3′. For large quantity validation, we developed species-specific primers that can distinguish *L. sumatrensis* and *L. uyii*. The primer sequences are; *L. sumatrensis*-specific *COI* forward primer: 5′- CCTATTATAATTGGAGGCTTT -3′, *L. sumatrensis*-specific *COI* reverse primer: 5′-CTACTGAAGGTCCTGCGTGT -3′, *L. uyii*-specific *COI* forward primer: 5′- CCCATTATAATCGGAGGTTTC -3′, *L. uyii*-specific *COI* reverse primer: 5′- CTACTGAGGGTCCTGCATGA -3′. PCR was run with 20 ng genomic DNA, 0.5 µM primers and KAPA2G Robust PCR Kit (Kapa Biosystems) according to a kit-provided standard protocol with annealing temperature at 58 °C and 35 cycles, followed by 3% agarose-gel electrophoresis.

### Quantitative analysis of reproductive anatomy

Dissection, measurements of somatic as well as reproductive indices and counting spermatangium numbers were carried out as previously described^28^. Briefly, the squid specimens were measured (mostly within one day after fishing) for dorsal mantle length (ML), body weight (BW), accessory gland weight (ACC), testis weight (TW), relative testis weight (RTW), ovary weight (OW), relative ovary weight (ROW), number of insemination sites used (SITE) and number of spermatangium attached to the female. To measure somatic weight, gonad weight (OW + ACC) was subtracted from total body weight. To count the number of spermatangia, the specimens were 10% formalin fixed, trimmed, and dissected under a stereomicroscope except that female buccal membranes were handled without fixation due to better visibility of attached spermatangia. ROW was calculated as 100 × OW × BW^−1^ and relative testis mass was calculated as 100 × TW × BW^−1^. Sperm were retrieved from the spermatangia attached to the females (unfrozen specimens), fixed with 10% formalin-containing seawater, photographed under a microscope (Nikon TE-2000) and thereafter sperm lengths were measured with Image J 1.52q (NIH, USA).

### Development of microsatellite markers

Genomic DNAs were purified from testes (wet weight of ~ 20 mg) of 30 representative mature males with kits; (QIAGEN Genomic-tip 20/G and TAKARA NucleoSpin® Tissue) according to manufacture protocols, verified their degradation levels with 0.8% agarose gel electrophoresis followed by visualization with ethidium bromide, and quantified their yield and quality with a micro-volume spectrophotometer and stored at − 80 °C. Short-read whole genome sequencing (150 bp paired-end, Novaseq6000/PE150, Novogene) was carried out, which yielded a total of 25.7 million clean reads (97.5% of raw reads) that were thereafter merged with PEAR (https://cme.h-its.org/exelixis/web/software/pear/doc.html), resulting in 3.19 million overlapped paired-end reads. The row reads were registered in the DNA Data Bank of Japan (DDBJ) Sequence Read Archive under accession number: DRA015757 (Submission), PRJDB15292 (BioProject), SAMD00579664 (BioSample) and DRX430961 (Experiment). Next, the sequence data were uploaded to Galaxy/NAAC to search for simple sequence repeat (SSR) with a MISA + Primer 3 pipeline, which detected a total of 22,298 SSRs. For an initial PCR test, 20 SSRs were selected at random and non-labelled primers synthesized. PCR was carried out with a kit (Platinum™ Direct PCR Universal Master Mix) and a thermal cycler (MiniAmp, Thermo Fisher Scientific) at optimized conditions: 20 ng of genomic DNA, 0.2 µM paired primers and PCR reaction consisting of an initial denaturing step of 94 °C for 2 min, then 40 cycles of 94 °C for 15 s, 56 °C for 15 s and 68 °C for 20 s followed by a final extension of 68 °C for 5 min. Amplicons with genomic DNAs from 10 male individuals were subjected to run on 8% mini-slab polyacrylamide gel electrophoresis to verify apparent variability in size. Thereafter, four primer sets were validated temporally and fluorescence (Hex, Fam, Cy3 and Ros)-tagged forward primers synthesized. Fragment length analysis (ABI PRISM 3130xl Genetic Analyzer) was performed with GeneScan™ 600 LIZ dye Size Standard (Thermo Fisher Scientific). Subsequently, the four selected microsatellite loci were fully characterized by open-source tools, OSIRIS (National Institutes of Health) and GenAlEx v.6.5.1 ^49^.

### Paternity analysis and DNA barcoding of spermatangia

Under a stereomicroscope, the female tissues that contain attached spermatangia were dissected out and fixed in 70% ethanol for 1 h, thereafter every single spermatangium removed with fine forceps from the tissue was placed carefully into the bottom of each well of the 96-well plate. To each well, 10 µl of lysis buffer containing 0.1 mg/ml protease-K (Direct PCR Master mix kit, Thermo Fisher Scientific) was added, followed by a 30-min incubation at 52 °C and heat (95 °C) inactivation for 1 min. The plates were kept frozen at -20 °C until use. Occasionally, the lysates in plates were spun down to precipitate undigested debris immediately prior to use. The DNA fragment length analysis was performed to genotype the spermatangia, where the PCR condition was same as described in the previous section of “SSR development” except that multiplex PCR was carried out. In addition, to identify the species of spermatangia attached to females, DNA sequencing of mitochondrial genome was done with cephalopod-specific universal primers^50^ and the SuperDye Direct Cycle Sequencing Kit (Thermo Fisher Scientific) system followed by Sanger sequencing with ABI PRISM 3130xl Genetic Analyzer.

### Evaluation of the costs/benefits of utilization of each insemination site

We assumed two hypothetical conditions: (1) if the time until spawning was short (a common strategy adopted by consort squid), insemination sites proximal to the egg deposition site would have higher fertilization success; and (2) if the time until spawning was long (a common strategy adopted by sneakers), insemination sites proximal to the sperm storage site (the seminal receptacle) would have higher fertilization success.

### Statistical data analysis

The statistical data analyses were performed with JMP Pro software, version 17.0.0 and SPSS software, version 23.0. The parametric assumptions were met for the statistical analysis. Generalized Linear Mixed Models (GLMM) with poisson distribution and log link function and Linear Mixed Models (LMM) were fit with sample ID as well as fishing days as random effects to analyze the attached spermatangium quantity per insemination sites, size variations of sperm among the different sites, site-dependent multiple paternity, and relative ovary weight of females using various number of sites for insemination. Additionally, Generalized Linear Model (GLM) was used to explore the variations of spermatangium number attached at each site with the fishing days. Tukey’s Kramer test was also used for pairwise comparison of the means and determine their significant differences (*P* < 0.05). Furthermore, multiple regression models were used to investigate the effects of spermatangium quantity at one site on the insemination at other sites and to know the influence of female growth and maturity status on the number of spermatangia at each insemination site. The frequency distribution of mantle length of adult males and females was performed to determine the presence or absence of their size dimorphism.

### Supplementary Information

Supplementary Information 1.

Supplementary Information 2.

## Data Availability

The entire dataset used for the measurements of somatic index, sperm size and SSR development are available from the Dryad data repository at 10.5061/Dryad.08kprr57x. GenBank accession numbers for microsatellite makers are LC756201, LC756202, LC756203 and LC756204. The row reads obtained by short-read whole genome sequencing were registered in the DNA Data Bank of Japan (DDBJ) Sequence Read Archive under accession number: DRA015757 (Submission), PRJDB15292 (BioProject), SAMD00579664 (BioSample) and DRX430961 (Experiment). Source data for underlying the graphs and plots in the main figures are provided in electronic supplementary materials.
