# Supplementary material for: Males conditionally inseminate at three female body locations according to female mating history and female maturity status in a squid
Source: Sci Rep. 2024 May 22;14:11702. doi: 10.1038/s41598-024-62062-7 (PMC11111733; doi:10.1038/s41598-024-62062-7)
Supplement: Supplementary file 1 — Supplementary Information 1. [file 41598_2024_62062_MOESM1_ESM.docx]

**Electronic Supplementary Materials**

**Supplementary tables**

**Males conditionally inseminate at three female body locations according to female mating history and female maturity status in a squid**

Kamrun Naher Azad, Md. Nur E. Alam, Makoto Nagata, Satoshi Tomano, Hiroki Ono, Kyoko Sugai, Noritaka Hirohashi

| Sex | year | dd-m | Number of individuals | %Male/  %Female | ML (mm) | | BW(g) | | Acc mg) | | Testis/  Ovary (mg) | | TSI/OSI | |
| --- | --- | --- | --- | --- | --- | --- | --- | --- | --- | --- | --- | --- | --- | --- |
|  |  |  |  |  | ave | std | ave | std | ave | std | ave | std | ave | std |
| Male | 2021 | 13-Jul | 51 | 42.15 | 78.8 | 6.14 | 20.5 | 10.7 | 335 | 124 | 634 | 110 | 3.1 | 0.76 |
|  | 2021 | 23-Jul | 55 | 45.45 | 81.3 | 4.9 | 18.9 | 2.46 | 360 | 88 | 610 | 134 | 3.23 | 0.71 |
|  | 2021 | 30-Jul | 24 | 55.81 | 80.8 | 6.42 | 19.6 | 5.58 | 373 | 150 | 614 | 89 | 3.13 | 0.42 |
|  | 2021 | 13-Sep | 17 | 25.37 | 76.2 | 8.51 | 15.7 | 3.85 | 253 | 68.4 | 402 | 89.1 | 2.57 | 0.59 |
|  | 2022 | 30-Jun | 62 | 51.67 | 66.3 | 6.96 | 11.6 | 2.73 | 213 | 129 | 407 | 138 | 3.51 | 0.85 |
|  | 2022 | 6-Jul | 75 | 42.86 | 72.9 | 5.21 | 15.2 | 2.62 | 261 | 86.4 | 526 | 105 | 3.46 | 0.98 |
|  | 2022 | 15-Jul | 29 | 25.44 | 76.4 | 8.87 | 17.2 | 3.91 | 282 | 114 | 545 | 156 | 3.17 | 0.66 |
|  | 2022 | 26-Jul | 84 | 53.85 | 73.5 | 7.11 | 17.3 | 3.82 | 297 | 128 | 478 | 126 | 2.76 | 0.67 |
|  |  | Total | 397 | average | 75.8 | 6.76 | 17 | 4.46 | 297 | 111 | 527 | 118 | 3.12 | 0.71 |
| Female | 2021 | 13-Jul | 70 | 57.85 | 86.3 | 6.68 | 26.7 | 5.79 | 3150 | 1487 | 2318 | 1197 | 8.69 | 4.49 |
|  | 2021 | 23-Jul | 66 | 54.55 | 88.6 | 10.1 | 27.5 | 5.28 | 3038 | 1262 | 2367 | 1007 | 8.59 | 3.66 |
|  | 2021 | 30-Jul | 19 | 44.19 | 87.8 | 10.1 | 27.4 | 6.72 | 3514 | 915 | 2287 | 644 | 8.34 | 2.35 |
|  | 2021 | 13-Sep | 50 | 74.63 | 78.1 | 6.48 | 21.1 | 3.77 | 2649 | 684 | 1712 | 470 | 8.12 | 2.23 |
|  | 2022 | 30-Jun | 58 | 48.33 | 67.8 | 8.31 | 13.2 | 4.36 | 601 | 776 | 462 | 639 | 3.51 | 4.85 |
|  | 2022 | 6-Jul | 100 | 57.14 | 75.3 | 6.45 | 18.6 | 3.82 | 1102 | 975 | 825 | 774 | 4.44 | 4.16 |
|  | 2022 | 15-Jul | 85 | 74.56 | 82.5 | 6.29 | 23.4 | 4.51 | 1965 | 1099 | 1417 | 940 | 6.06 | 4.02 |
|  | 2022 | 26-Jul | 72 | 46.15 | 77.6 | 7.62 | 21.4 | 4.88 | 1480 | 2593 | 742 | 863 | 3.47 | 4.03 |
|  |  | Total | 520 | average | 80.5 | 7.75 | 22.4 | 4.89 | 2187 | 1224 | 1516 | 817 | 6.4 | 3.72 |
| **Male & Female** |  | **Sum** | **917** |  | | | | | | | | | | |

**Electronic supplementary material, Table S1 Summary of individual measurements for growth, maturity and sperm insemination sites**

|  | | Number of females having spermatangia at insemination sites | | | | | % females | | | | | |
| --- | --- | --- | --- | --- | --- | --- | --- | --- | --- | --- | --- | --- |
| year | dd-m | none | BM | BM &  ARM | BM &  ARM &  EYE | BM &  EYE | total | none | BM | BM & ARM | BM & ARM & EYE | BM & EYE |
| 2021 | 13-Jul | 14 | 4 | 21 | 30 | 1 | 70 | 20 | 5.71 | 30 | 42.9 | 1.43 |
| 2021 | 23-Jul | 11 | 10 | 22 | 23 | 0 | 66 | 16.7 | 15.2 | 33.3 | 34.8 | 0 |
| 2021 | 30-Jul | 0 | 0 | 9 | 10 | 0 | 19 | 0 | 0 | 47.4 | 52.6 | 0 |
| 2021 | 13-Sep | 0 | 0 | 4 | 46 | 0 | 50 | 0 | 0 | 8 | 92 | 0 |
| 2022 | 30-Jun | 45 | 2 | 4 | 6 | 1 | 58 | 77.6 | 3.45 | 6.9 | 10.3 | 1.72 |
| 2022 | 6-Jul | 69 | 1 | 13 | 17 | 0 | 100 | 69 | 1 | 13 | 17 | 0 |
| 2022 | 15-Jul | 30 | 3 | 21 | 31 | 0 | 85 | 35.3 | 3.53 | 24.7 | 36.5 | 0 |
| 2022 | 26-Jul | 46 | 1 | 4 | 21 | 0 | 72 | 63.9 | 1.39 | 5.56 | 29.2 | 0 |

ML: mantle length, BW: body weight. ACC: accessory reproductive gland weight, TSI: testicular somatic index, OSI: ovarian somatic index

**Electronic supplementary material, Table S2 Paternity identification and paternity sharing of the spermatangia stored at three different insemination sites within a female**

| **female #01** | | | | | | | |
| --- | --- | --- | --- | --- | --- | --- | --- |
|  | | | | **Number of spermatangia** | | | |
| Insemination site | | | | BM | ARM | EYE | Combined (%) |
| Total spermatangia attached | | | | 12 | 111 | 129 | 252 (100) |
| Genotyping unexamined or failed | | | | 5 | 63 | 50 | 118 (46.8) |
| Genotyping succeded | | | | 7 | 48 | 79 | 134 (53.2) |
|  | SSR locus | | |  | | | |
| Genotype | Lu1738 | Lu3099 | Lu4288 | 5 | 63 | 50 |  |
| # 1 sire | 137/140 | 148/157 | 166/175 | 6 | 16 | 60 | 82 |
| # 2 sire | 161/173 | 136/145 | 169/175 | 0 | 24 | 14 | 38 |
| # 3 sire | 119/119 | 115/145 | 166/175 | 0 | 5 | 0 | 5 |
| # 4 sire | 149/164 | 148/157 | 154/178 | 0 | 0 | 5 | 5 |
| # 5 sire | 161/164 | 154/160 | 172/171 | 1 | 1 | 0 | 2 |
| # 6 sire | 167/173 | 136/145 | 169/175 | 0 | 1 | 0 | 1 |
| # 7 sire | 158/167 | 139/157 | 154/166 | 0 | 1 | 0 | 1 |
| Number of sires | | | | 2 | 6 | 3 | 7 |

| **female #02** | | | | | | | |
| --- | --- | --- | --- | --- | --- | --- | --- |
|  | | | | **Number of spermatangia** | | | |
| Insemination site | | | | BM | ARM | EYE | Combined (%) |
| Total spermatangia attached | | | | 28 | 80 | 205 | 313 (100_ |
| Genotyping unexamined or failed | | | | 22 | 10 | 106 | 138 (33.8) |
| Genotyping succeded | | | | 6 | 70 | 99 | 175 (66.2) |
|  | SSR locus | | |  | | | |
| Genotype | Lu1738 | Lu3099 | Lu4288 | 22 | 10 | 106 |  |
| # 1 sire | 143/143 | 136/142 | 184/184 | 5 | 35 | 73 | 113 |
| # 2 sire | 140/152 | 145/154 | 163/178 | 1 | 33 | 18 | 52 |
| # 3 sire | 161/173 | 136/145 | 169/175 | 0 | 0 | 2 | 2 |
| # 4 sire | 140/140 | 157/157 | 166/175 | 0 | 0 | 2 | 2 |
| # 5 sire | 116/119 | 145/145 | 169/214 | 0 | 1 | 0 | 1 |
| # 6 sire | 158/158 | 139/142 | 166/190 | 0 | 1 | 0 | 1 |
| # 7 sire | 149/164 | 139/142 | 154/178 | 0 | 0 | 1 | 1 |
| # 8 sire | 137/155 | 145/148 | 175/184 | 0 | 0 | 1 | 1 |
| # 9 sire | 140/152 | 142/157 | 160/169 | 0 | 0 | 1 | 1 |
| Number of sires | | | | 2 | 4 | 7 | 9 |

| **female #03** | | | | | | | |
| --- | --- | --- | --- | --- | --- | --- | --- |
|  | | | | **Number of spermatangia** | | | |
| Insemination site | | | | BM | ARM | EYE | Combined (%) |
| Total spermatangia attached | | | | 97 | 238 | 200 | 535 (100) |
| Genotyping unexamined or failed | | | | 52 | 155 | 120 | 327 (61.1) |
| Genotyping succeded | | | | 47 | 83 | 80 | 210 (38.9) |
|  | SSR locus | | |  | | | |
| Genotype | Lu1738 | Lu3099 | Lu4288 | 52 | 155 | 120 |  |
| # 1 sire | 143/167 | 148/163 | 172/175 | 0 | 32 | 64 | 96 |
| # 2 sire | 167/167 | 151/157 | 172/181 | 36 | 27 | 0 | 63 |
| # 3 sire | 143/143 | 136/160 | 172/175 | 10 | 16 | 16 | 42 |
| # 4 sire | 167/167 | 145/151 | 172/181 | 1 | 1 | 0 | 2 |
| # 5 sire | 158/158 | 142/148 | 175/202 | 0 | 2 | 0 | 2 |
| # 6 sire | 116/116 | 142/145 | 166/166 | 0 | 2 | 0 | 2 |
| # 7 sire | 137/155 | 145/148 | 187/187 | 0 | 1 | 0 | 1 |
| # 8 sire | 143/143 | 136/136 | 172/214 | 0 | 1 | 0 | 1 |
| # 9 sire | 164/164 | 142/151 | 166/172 | 0 | 1 | 0 | 1 |
| Number of sires | | | | 3 | 9 | 2 | 9 |

Each SSR locus shows allele sizes (bp/bp) of identified sires.

**Elactronic supplementary material, Table S3 The hypothetical rule of a set order in a male insemination preference**

Suppose there is a set order in which the insemination site is preferentially used first by males. Suppose further that males prefer to inseminate first at X followed by Y, then Z, which is defined as (X, Y, Z). In this rule, there are three possible cases of female status: only X is used; both X and Y are used; and all X, Y, and Z are used. In any other case, they are regarded as "against the rule.”

For all possible combinations of (X, Y, Z), the frequency (% in total) of each sequential event that could occur in the collected specimens (n = 304) is as follows. "others" indicate the frequencies of exceptions (against the rule).

|  | inseminated at | | | |
| --- | --- | --- | --- | --- |
| (X, Y, Z) | X | X, Y | X, Y, Z | others |
| (EYE, ARM, BM)  (EYE, BM, ARM)  (ARM, BM, EYE)  (ARM, EYE, BM)  (BM, EYE, ARM)  (BM, ARM, EYE) | *0.000 %*  *0.000 %*  *0.000 %*  *0.000 %*  *6.908 %*  *6.908 %* | *0.329 %*  *0.329 %*  *0.329 %*  *32.24 %*  *0.329 %*  *32.24 %* | *60.20 %*  *60.20 %*  *60.20 %*  *60.20 %*  *60.20 %*  *60.20 %* | *39.47 %*  *39.47 %*  *39.47 %*  *7.566 %*  *32.57 %*  *0.658 %* |

A low frequency (>1%) of other cases (frequency of exceptions) was observed only in (BM, ARM, EYE). If the rule is applied strictly, then it is most appropriate that a set order of initial use for insemination is BM$\to$ARM$\to$EYE.

**Electronic supplementary material, Table S4 Statistical analysis by rank cases of spermatangia insemination at female body sites**

| Insemination of spermatangia | R_Insemination | R_AN001 |
| --- | --- | --- |
| BM | 13 | 1 |
| BM & ARM | 72 | 2 |
| BM & ARM & EYE | 214 | 3 |

R_Insemination, Mean Rank of tied values; R_AN001, Consecutive Ranks of ties sharing the same value.

Ranks are in ascending order.

**Electronic supplementary material, Table S5 Effects of female growth/maturity indices and site usage on the number of spermatangia attached to each site or all sites**

| Site | ML | | ACC | | OW | | BW | | SITE | |  |
| --- | --- | --- | --- | --- | --- | --- | --- | --- | --- | --- | --- |
|  | β_ML_ | *P* | β_ACC_ | *P* | β_OW_ | *P* | β_BW_ | *P* | β_SITE_ | *P* | R^2^ |
| TOTAL | 0.076 | *0.381* | 0 | *0.997* | **0.340** | *0.000* | -**0.293** | *0.011* | **0.529** | *0.000* | **0.34** |
| BM | -0.056 | *0.600* | -0.025 | *0.741* | 0.174 | *0.109* | 0.077 | *0.585* | 0.199 | *0.842* | 0.02 |
| ARM | 0.035 | *0.737* | 0.026 | *0.725* | **0.452** | *0.000* | **-0.320** | *0.022* | **0.188** | *0.004* | **0.13** |
| EYE | 0.234 | *0.195* | 0.01 | *0.104* | **0.283** | *0.040* | **-0.547** | *0.016* | 0.038 | *0.660* | 0.02 |

Multiple regression of female growth (ML, mantle length; BW, body weight) and maturity (ACC, accessory gland weight; OW, ovary weight; site usage (SITE, Number of insemination sites used) indices on the number of spermatangia at each site (BM, ARM and EYE) or all sites (TOTAL). Significant regression coefficients are indicated in bold.

**Supplementary figures**

**A**

Query: Loliolus uyii voucher OUC00236 cytochrome c oxidase subunit I (COX1) gene, partial cds; mitochondrial Query ID: OL425817.1 Length: 653

Query range 1: 1 to 120

Query:L.uyii1 TTGGATTTGAGCAGGATTAGTTGGTACATCATTAAGCCTTATAATTCGAACAGAGTTAGGTAAACCAGGTTCACTTCTAAATGATGATCAATTATACAATGTAGTAGTAACTGCTCACGG 120

uyii 1 ........................................................................................................................ 120

uyii 1 ........................................................................................................................ 120

uyii 1 ........................................................................................................................ 120

uyii 1 ........................................................................................................................ 120

sumatrensis 1 ......T......................................A........G.....A..........................T....................... 111

sumatrensis 1 ......T......................................A........G.....A..........................T....................... 111

sumatrensis 1 ......T......................................A........G.....A..........................T....................... 111

sumatrensis 1 ......T......................................A........G.....A..........................T....................... 111

beka 1 18 ...........................T........T.................A.....A........A.....C...............C.........................T.. 138

beka 1 18 ...........................T........T.................A.....A........A.....C........C......C.........................T.. 138

beka 1 18 ...........................T........T.................A.....A........A.....C........C......C.........................T.. 138

beka 1 18 ...........................T........T.................A.....A........A.....C........C......C.........................T.. 138

japonica 1 ............G.....A.....T........T.................A.....G........A.....C.....C.....C...C....T.......................... 117

japonica 1 8896 ...........G.....A.....T........T.................A.....G........A.....C.....C.....C...C....T....................... 9011

japonica 1 1 .....G.....A.....T........T.................A.....G........A.....C.....C.....C...C....T....................... 110

japonica 1 3 ...G.....A.....T........T.................A.....G........A.....C.....C.....C...C....T....................... 110

Query:L.uyii121 TTTTATTATAAttttttttATAGTTATACCCATTATAATCGGAGGTTTCGGAAACTGACTAGTACCTTTGATACTTGGAGCACCTGATATGGCCTTTCCACGTATAAATAATATAAGTTT 240

uyii 121 ........................................................................................................................ 240

uyii 121 ........................................................................................................................ 240

uyii 121 ........................................................................................................................ 240

uyii 121 ........................................................................................................................ 240

sumatrensis 112 ..............................T........T.....C..T.........T.G........A........T..G..G........T..............C........... 231

sumatrensis 112 ..............................T........T.....C..T.........T.G........A........T..G..G........T..............C........... 231

sumatrensis 112 ..............................T........T.....C..T.........T.G........A........T..G..G........T..............C........... 231

sumatrensis 112 ..............................T........T.....C..T.........T.G........A........T..G..G........T..............C........... 231

beka 1 139 ..............................T...........G..G..T.....T...T..........A..............A.....A.....C....................A.. 258

beka 1 139 ..............................T...........G..G..T.....T...T..........A..............A.....A.....C....................A.. 258

beka 1 139 ..............................T...........G..G..T.....T...T..........A..............A.....A.....C....................A.. 258

beka 1 139 ..............................T...........G..G..T.....T...T..........A..............A.....A.....C....................A.. 258

japonica 1 118 ..............................T........T..G..G..T.....T..............A..............A.....A.....C...........C........... 237

japonica.1 9012 ..............................T........T..G..G..T.....T..............A..............A.....A.....C...........C........... 9131

japonica 1 111 ..............................T........T..G..G..T.....T..............A..............A.....A.....C...........C........... 230

japonica 1 111 ..............................T........T..G..G..T.....T..............A..............A.....A.....C...........C........... 230

Query:L.uyii241 CTGATTACTTCCCCCCTCATTAACACTACTATTAGCATCTTCCGCAGTTGAAAGAGGAGCAGGTACAGGCTGAACAGTTTACCCCCCTTTATCCAGCAACCTATCTCATGCAGGACCCTC 360

uyii 241 ........................................................................................................................ 360

uyii 241 ........................................................................................................................ 360

uyii 241 ........................................................................................................................ 360

uyii 241 ........................................................................................................................ 360

sumatrensis 232 ............A............T....................................................A.....T..............T.....A..C........T.. 351

sumatrensis 232 ............A............T....................................................A.....T..............T.....A..C........T.. 351

sumatrensis 232 ............A............T....................................................A.....T..............T.....A..C........T.. 351

sumatrensis 232 ............A............T....................................................A.....T..............T.....A..C.....G..T.. 351

beka 259 ....................................C..C..A.....A........G........T..G........A..T..T........T..T..T..C..A.....G........ 378

beka 259 ....................................C..C..A.....A........G........T..G........A..T..T........T..T..T..C..A.....G........ 378

beka 259 ....................................C..C..A.....A........G........T..G........A..T..T........T..T..T..C..A.....G........ 378

beka 259 ....................................C..C..A.....A........G........T..G........A..T..T........T..T..T..C..A.....G........ 378

japonica 238 ....C.......A...........T...T.......C.....A.....C..G.....G........C..A........A.....T........T..T..T..T..............T.. 357

japonica 9132 ....C.......A...........T...T.......C.....A.....C..G.....G........C..A........A.....T........T..T..T..T..............T.. 9251

japonica 231 ....C.......A...........T...T.......C.....A.....C..G.....G........C..A........A.....T........T..T..T..T..............T.. 350

japonica 231 ....C.......A...........T...T.......C.....A.....C..G.....G........C..A........A.....T........T..T..T..T..............T.. 350

Query:L.uyii361 AGTAGATCTCGCTATTTTCTCATTACATTTAGCTGGTATTTCTTCTATTTTAGGAGCTATTAACTTTATCACAACCATTATAAATATACGTTGAGAAGGACTCCTAATAGAACGAATATC 480

uyii 361 ........................................................................................................................ 480

uyii 361 ........................................................................................................................ 480

uyii 361 ........................................................................................................................ 480

uyii 361 ........................................................................................................................ 480

sumatrensis 352 .........................................................C.....T....................C..G............T.A................. 471

sumatrensis 352 .........................................................C.....T.................G..C..G............T.A................. 471

sumatrensis 352 .........................................................C.....T.................G..C..G............T.A................. 471

sumatrensis 352 .........................................................C.....T.................G..C..G............T.A................. 471

beka 379 ...T..C..A........T.........C....................C.......A.....T.....T..T.....C...........C...........AT................ 498

beka 379 ...T..C..A........T.........C....................C.......A.....T.....T..T.....C...........C...........AT................ 498

beka 379 ...T..C..A........T.........C....................C.......A.....T.....T..T.....C.......................AT................ 498

beka 379 ...T..C..A........T.........C....................C.......A.....T.....T..T.....C...........C...........AT................ 498

japonica 358 ...T..C..A........T...C.T..C..............A......C.......A.....T.....T.....T..........................AT..........T..... 477

japonica 9252 ...T..C..A........T...C.T..C..............A......C.......A.....T.....T.....T..........................AT..........T..... 9371

japonica 351 ...T..C..A........T...C.T..C..............A......C.......A.....T.....T.....T..........................AT..........T..... 470

japonica 351 ...T..C..A........T...C.T..C..............A......C.......A.....T.....T.....T..........................AT..........T..... 470

The sequences highlighted are used for Loliolus-specific universal primers (*cyan*) and species-specific primers (*yellow*).

**B**

| Nucleotide position | 151 | 160 | 166 | 169 | 179 | 181 | 190 | 199 | 202 | 205 | 214 | 229 | 253 | 266 |
| --- | --- | --- | --- | --- | --- | --- | --- | --- | --- | --- | --- | --- | --- | --- |
| *L. uyii* COI | **C** | **C** | **T** | **C** | **C** | **A** | **G** | **A** | **A** | **T** | **C** | **T** | **C** | **C** |
| *L. sumatrensis* COI | **T** | **T** | **C** | **T** | **T** | **G** | **A** | **T** | **G** | **G** | **T** | **C** | **A** | **T** |
| Testis1 | T | T | C | T | T | G | A | T | G | G | T | C | A | T |
| Testis2 | T | T | C | N | T | G | A | T | G | G | T | C | A | T |
| Testis3 | T | T | C | T | T | G | A | T | G | G | T | C | A | T |
| Testis4 | T | T | C | N | T | G | A | T | G | G | T | C | A | T |
| Testis5 | T | T | C | T | T | G | A | T | G | G | T | C | A | T |
| Testis6 | T | T | C | T | T | G | A | T | G | G | T | C | A | T |
| Testis6 | T | T | C | T | T | G | A | T | G | G | T | C | A | T |
| Testis8 | T | N | C | T | T | G | A | T | G | G | T | C | A | T |
| Testis9 | T | T | C | T | T | G | A | T | G | G | T | C | A | T |
| Testis10 | T | T | C | T | T | G | A | T | G | G | T | C | A | T |
| Testis11 | T | T | C | T | T | G | A | T | G | G | T | C | A | T |
| Testis12 | T | T | C | T | T | G | A | T | G | G | T | C | A | T |
| Testis13 | T | T | C | T | T | G | A | T | G | G | T | C | A | T |
| Testis14 | T | T | C | T | T | G | A | T | G | G | T | C | A | T |
| Testis15 | T | T | C | T | T | G | A | T | G | G | T | C | A | T |
| Testis16 | T | T | C | T | T | G | A | T | G | G | T | C | A | T |
| Testis17 | T | T | C | T | T | G | A | T | G | G | T | C | A | T |
| Testis18 | T | T | C | T | T | G | A | T | G | G | T | C | A | T |
| Testis19 | T | T | C | T | T | G | A | T | G | G | T | C | A | T |
| Testis20 | T | T | C | T | T | G | A | T | G | G | T | C | A | T |
| Testis21 | T | T | C | T | T | G | A | T | G | G | T | C | A | T |
| Testis22 | T | T | C | T | T | G | A | T | G | G | T | C | A | T |
| Testis23 | T | N | C | T | T | G | A | T | G | G | T | C | A | T |
| Testis24 | T | N | C | T | T | G | A | T | G | G | T | C | A | T |
| Testis25 | T | T | C | T | T | G | A | T | G | G | T | C | A | T |
| Testis26 | T | T | C | T | T | G | A | T | G | G | T | C | A | T |
| Testis27 | T | N | C | T | T | G | A | T | G | G | T | C | A | T |
| Testis28 | T | T | C | T | T | G | A | T | G | G | T | C | A | T |
| Testis29 | T | T | C | T | T | G | A | T | G | G | T | C | A | T |
| Testis30 | T | T | C | T | T | G | A | T | G | G | T | C | A | T |
| Testis31 | T | T | C | T | T | G | A | T | G | G | T | C | A | T |
| Testis32 | T | T | C | N | T | G | A | T | G | G | T | C | A | T |
| Testis33 | T | T | C | T | T | G | A | T | G | G | T | C | A | T |
| Testis34 | T | T | C | T | T | G | A | T | G | G | T | C | A | T |
| Testis35 | T | T | C | T | T | G | A | T | G | G | T | C | A | T |
| Testis36 | T | T | C | T | T | G | A | T | G | G | T | C | A | T |
| Testis37 | T | N | C | T | T | G | A | T | G | G | T | C | N | T |
| Testis38 | T | T | C | T | T | G | A | T | G | G | T | C | A | T |
| Testis39 | T | T | C | T | T | G | A | T | G | G | T | C | A | T |
| Testis40 | T | T | C | T | T | G | A | T | G | G | T | C | A | T |
| Testis41 | T | T | C | N | T | G | A | T | G | G | T | C | A | T |
| Testis42 | T | T | C | T | T | G | A | T | G | G | T | C | A | T |
| Testis43 | T | T | C | T | T | G | A | T | G | G | T | C | A | T |
| Testis44 | T | T | C | T | T | G | A | T | G | G | T | C | A | T |
| Testis45 | T | T | C | T | T | G | A | T | G | G | T | C | A | T |

**C**

| Nucleotide position | 151 | 160 | 166 | 169 | 179 | 181 | 190 | 199 | 202 | 205 | 214 | 229 | 253 | 266 |
| --- | --- | --- | --- | --- | --- | --- | --- | --- | --- | --- | --- | --- | --- | --- |
| *L. uyii* COI | **C** | **C** | **T** | **C** | **C** | **A** | **G** | **A** | **A** | **T** | **C** | **T** | **C** | **C** |
| *L. sumatrensis* COI | **T** | **T** | **C** | **T** | **T** | **G** | **A** | **T** | **G** | **G** | **T** | **C** | **A** | **T** |
| Bm1 | T | T | C | T | T | G | A | T | G | G | T | C | A | T |
| Bm2 | T | N | C | T | T | G | A | T | G | G | T | C | A | T |
| Bm3 | T | T | C | T | T | G | A | T | G | G | T | C | A | T |
| Bm4 | T | T | C | T | T | G | A | T | G | G | T | C | A | T |
| Bm5 | T | T | C | T | T | G | A | T | G | G | T | C | A | T |
| Bm6 | T | T | C | T | T | G | A | T | G | G | T | C | A | T |
| Bm7 | T | T | C | T | T | G | A | T | G | G | T | C | A | T |
| Bm8 | T | T | C | T | T | G | A | T | G | G | T | C | A | T |
| Bm9 | T | T | C | T | T | G | A | T | G | G | T | C | A | T |
| Bm10 | N | T | C | T | T | G | A | T | G | G | T | C | A | T |
| Bm11 | T | T | C | T | T | G | A | T | G | G | T | C | A | T |
| Bm12 | T | T | C | T | T | G | A | T | G | G | T | C | A | T |
| Bm13 | T | T | C | T | T | G | A | T | G | G | T | C | A | T |
| Bm14 | T | T | C | T | T | G | A | T | G | G | T | C | A | T |
| Bm15 | T | T | C | T | T | G | A | T | G | G | T | C | A | T |
| Bm16 | T | T | C | T | T | G | A | T | G | G | T | C | A | T |
| Arm1 | T | T | C | T | T | G | A | T | G | G | T | C | A | T |
| Arm2 | T | T | C | T | T | G | A | T | G | G | T | C | A | T |
| Arm3 | T | T | C | T | T | G | A | T | G | G | T | C | A | T |
| Arm4 | T | T | C | T | T | G | A | T | G | G | T | C | A | T |
| Arm5 | T | T | C | T | T | G | A | T | G | G | T | C | A | T |
| Arm6 | T | T | C | T | T | G | A | T | G | G | T | C | A | T |
| Arm7 | T | T | C | T | T | G | A | T | G | G | T | C | A | T |
| Arm8 | T | T | C | T | T | G | A | T | G | G | T | C | A | T |
| Arm9 | T | T | C | T | T | G | A | T | G | G | T | C | A | T |
| Arm10 | T | T | C | T | T | G | A | T | G | G | T | C | A | T |
| Arm11 | T | T | C | T | T | G | A | T | G | G | T | C | A | T |
| Arm12 | T | T | C | T | T | G | A | T | G | G | T | C | A | T |
| Arm13 | T | T | C | T | T | G | A | T | G | G | T | C | A | T |
| Arm14 | T | T | C | T | T | G | A | T | G | G | T | C | A | T |
| Arm15 | T | T | C | T | T | G | A | T | G | G | T | C | A | T |
| Eye1 | T | T | C | T | T | G | A | T | G | G | T | C | A | T |
| Eye2 | T | T | C | T | T | G | A | T | G | G | T | C | A | T |
| Eye3 | T | T | C | T | T | G | A | T | G | G | T | C | A | T |
| Eye4 | T | T | C | T | T | G | A | T | G | G | T | C | A | T |
| Eye5 | T | T | C | T | T | G | A | T | G | G | T | C | A | T |
| Eye6 | T | T | C | T | T | G | A | T | G | G | T | C | A | T |
| Eye7 | T | T | C | T | T | G | A | T | G | G | T | C | A | T |
| Eye8 | T | T | C | T | T | G | A | T | G | G | T | C | A | T |
| Eye9 | T | T | C | T | T | G | A | T | G | G | T | C | A | T |
| Eye10 | N | N | N | N | T | G | A | T | G | G | N | C | A | T |
| Eye11 | T | T | C | T | T | G | A | T | G | G | T | C | A | T |
| Eye12 | T | T | C | T | T | G | A | T | G | G | T | C | A | T |
| Eye13 | T | T | C | T | T | G | A | T | G | G | T | C | A | T |

**D**

**
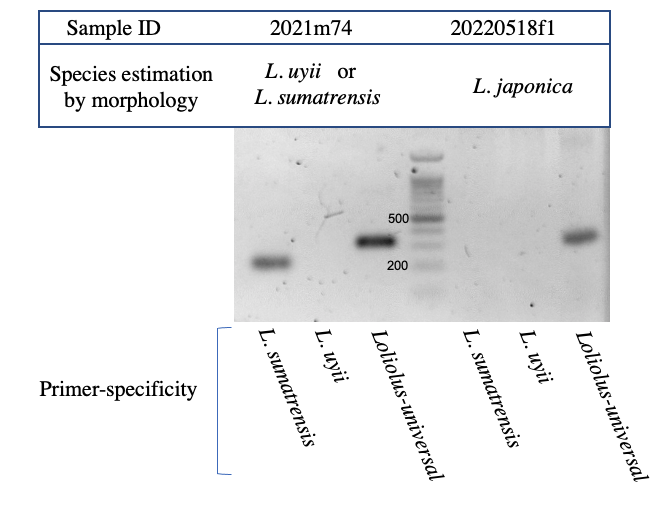
**

**Electronic supplementary material, Fig. S1 Assignment of study species by DNA barcoding and a PCR-based diagnosis**

# A, Shown are the alignment of mitochondrial *COI* sequences of *L. uyii, L. sumatrensis, L. beka and L. japonica* (four representative individuals per species) taken from the GenBank database. The sequences highlighted are used for Loliolus-specific universal primers (*cyan*) and species-specific primers (*yellow*). B, C, nucleotide comparisons of the DNA sequences at positions where *L. uyii* and *L. sumatrensis* are different. Testes from 45 males (B) and 44 spermatangia (16 from BM, 15 from ARM and 13 from EYE) from a female (C) were used to purifiy genomic DNAs for DNA barcording. D, A rapid PCR-based diagnosis assay was developed with use of species-spececific primer sets, by which morphologically similar *L. uyii* and *L. sumatrensis* were found distinguishable. Shown were representative results with genomic DNAs from *Loliolus s*quids collected in mid-summer (individual ID: 2021m74) and in early summer (individual ID: 20220518f1). Species callected in May was assingned morphologically as *L. japonica*. This assay revealed that all tested specimens (48 spermatangia from BM, 48 spermatangia from ARM, 48 spermatangia from EYE and testes from 48 males) collected at the Seto Inland Sea during July-September are found to be *L. sumatrensis.* N; not determined

#
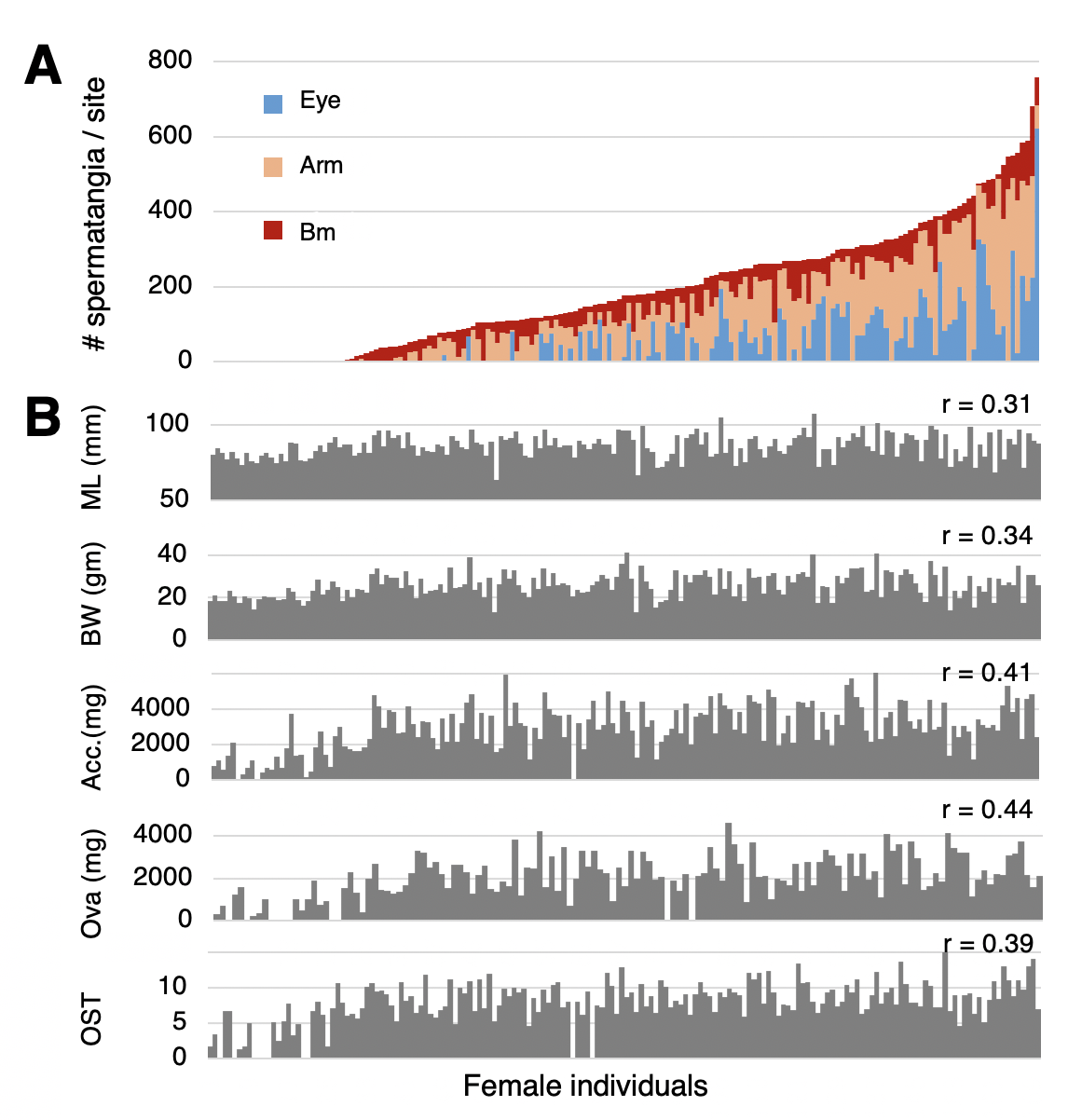


# Electronic supplementary material, Fig. S2 Individual variableness in total and site-dependent numbers of attached spermatangia and their least correlations with maturation and growth status.

# A, B; The stacked bars (A) showing the total number of spermatangia attached to three insemination sites per female, arranged in ascending order, in relation to individual growth and mature indices (B). Shown in *top right* of each graph represents the correlation coefficient (r) between total number of spermatangia/female and each index. ML: mantle length, BW: body weight, Acc: accessory gland weight, Ova: ovary weight, OST: relative ovary weight.

**
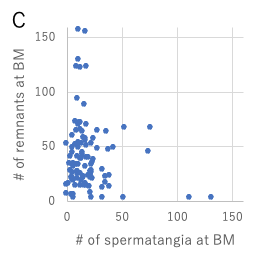
**

**Electronic supplementary material, Fig. S**
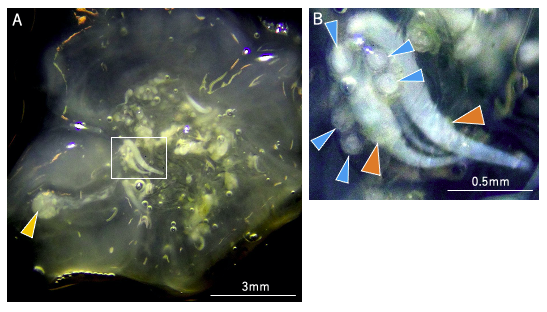
**3 A representative photograph of the isolated female buccal membrane with attached spermatangia and their remnants.**

A, Viewing from the surface of a whole buccal membrane with the seminal receptacle (*yellow arrowhead*). B, *Inset* showing intact spermatangia (*orange arrowhead*) and the remnants of the spermatangia (*blue arrowhead*). C, Individual variability in holding spermatangium number and remnant number at the buccal membrane. Each dot represents an individual female.
