# Supplementary figures and images for: Males conditionally inseminate at three female body locations according to female mating history and female maturity status in a squid
Source: Sci Rep. 2024 May 22;14:11702. doi: 10.1038/s41598-024-62062-7 (PMC11111733; doi:10.1038/s41598-024-62062-7)

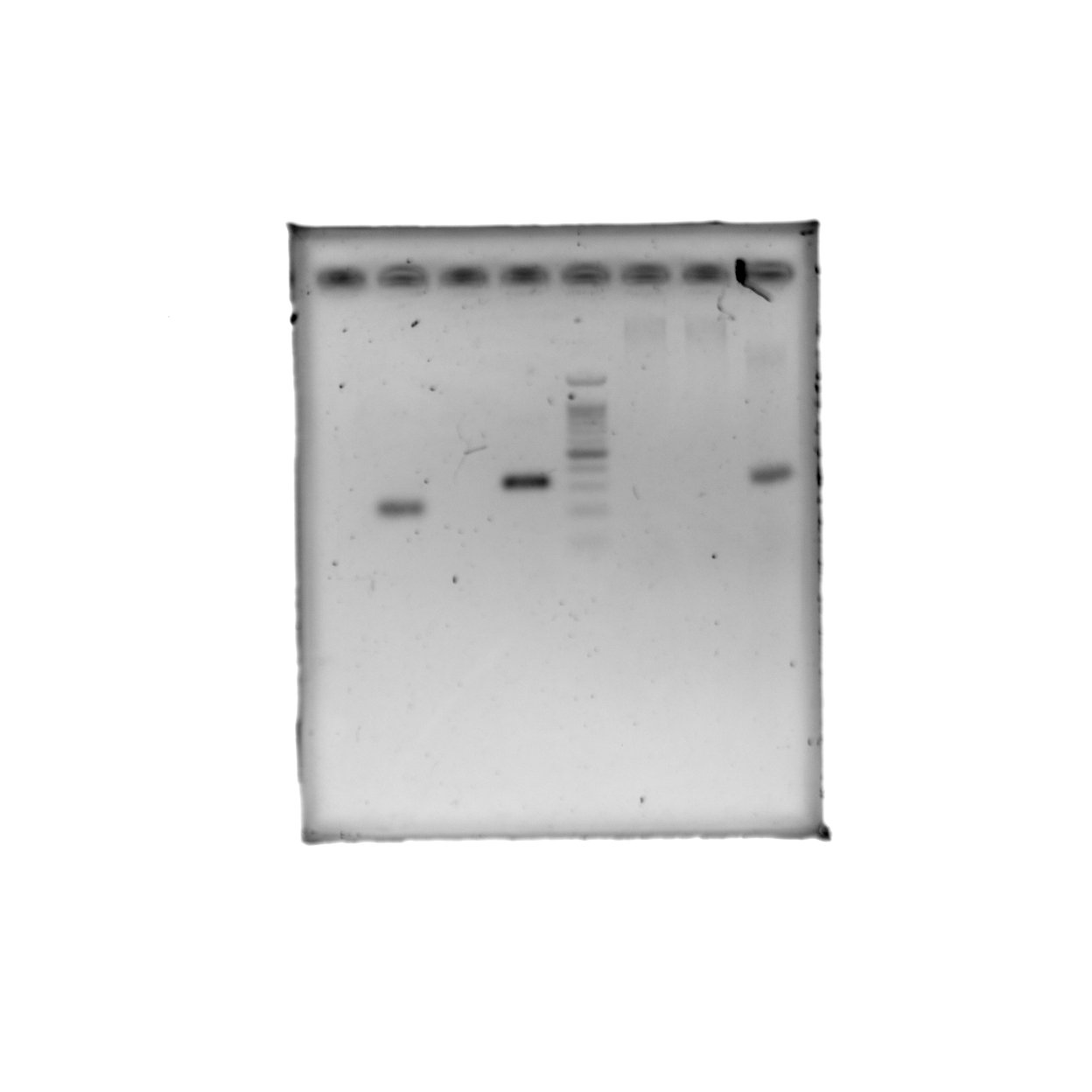

Supplement: Supplementary file 2 — Supplementary Information 2. [file 41598_2024_62062_MOESM2_ESM.jpg]
